# Gene Duplication Associated with Increased Fluconazole Tolerance in *Candida auris* cells of Advanced Generational Age

**DOI:** 10.1038/s41598-019-41513-6

**Published:** 2019-03-25

**Authors:** Somanon Bhattacharya, Thomas Holowka, Erika P. Orner, Bettina C. Fries

**Affiliations:** 1 Division of Infectious Diseases, Department of Medicine, Stony Brook University, Stony Brook, New York USA; 2 Department of Molecular Genetics and Microbiology, Stony Brook University, Stony Brook, New York USA; 3 Present Address: Department of Medicine, Yale School of Medicine, New Haven, Connecticut USA; 4 Veterans Admininistration Medical Center, Northport, New York USA

## Abstract

*Candida auris* is an emerging multi-drug resistant yeast that causes systemic infections. Here we show that *C*. *auris* undergoes replicative aging (RA) that results from asymmetric cell division and causes phenotypic differences between mother and daughter cells similar to other pathogenic yeasts. Importantly, older *C*. *auris* cells (10 generations) exhibited higher tolerance to fluconazole (FLC), micafungin, 5- flucytosine and amphotericin B compared to younger (0–3 generation) cells. Increased FLC tolerance was associated with increased Rhodamine 6G (R6G) efflux and therapeutic failure of FLC in a *Galleria* infection model. The higher efflux in the older cells correlated with overexpression of the efflux pump encoding gene *CDR1* (4-fold). In addition, 8-fold upregulation of the azole target encoding gene *ERG11* was noted in the older cells. Analysis of genomic DNA from older cells by qPCR indicates that transient gene duplication of *CDR1* and *ERG11* causes the observed age-dependent enhanced FLC tolerance in *C*. *auris* strains. Furthermore, older cells exhibited a thickened cell wall, decreased neutrophil killing (24% vs 50%), increased epithelial cell adhesion (31.6% vs 17.8%) and upregulation of adhesin protein Als5p. Thus, this study demonstrates that transient gene duplication can occur during RA, causing increased FLC tolerance in old *C*. *auris* cells.

## Introduction

*Candida auris* is an emerging multi-drug resistant yeast that is most closely related to *Candida lusitaniae*^1,2^ and emergence of distinct clones of *C*. *auris* have been described in different continents^2^. This yeast is smaller in cell size than other pathogenic yeasts causing nosocomial infections in patients with co-morbidities^3^, but their infections are associated with a high mortality rate (>50%). Therapeutic failures result from two important characteristics of this fungus. First, *C*. *auris* strains exhibit colonization that persists for a prolonged time. Second, *C*. *auris* isolated from infected patients are resistant to a broad spectrum of antifungals like Fluconazole (FLC), amphotericin B, flucytosine and even echinocandins^2,4–6^. However, little is known about the virulence traits of this fungus, with only a preliminary genomic sequence available to date^7^. Additionally, why this species is rapidly emerging worldwide with such a high prevalence of FLC resistance remains unclear. FLC resistance in *C*. *auris* has been associated with increased efflux activities and overexpression of *ERG11*^2,8^ similar to other *Candida* species. Prolonged use of fungistatic drug like FLC can select for FLC- tolerant cells from a fungal population promoting the recent emergence of this new fungal pathogen. Fungal cells of advanced generational age also exhibit increased tolerance to a variety of antifungals^9,10^, suggesting that generational aging may play a role in the virulence of *C*. *auris*.

Replicative life span (RLS) is the result of asymmetric cell division that causes phenotypic changes between the mother and the daughter cells. The pathogenic yeasts *Cryptococcus neoformans* and *Candida glabrata* exhibit a median RLS that is highly variable but reproducible among clinical strains^9,11^. Importantly, ensuing phenotypic changes in the aging mothers render yeast cells with advanced generational age (here referred to as “older”) with increased resistance to killing by the host phagocytes, and most importantly increased resistance to antifungal drug therapy^9,11^. Specifically, it was demonstrated that older *C*. *neoformans* and *C*. *glabrata* cells were significantly more tolerant to amphotericin B (AMB), echinocandin and fluconazole (FLC)^11–13^. This is relevant because *in vivo* considerable selection occurs within an expanding fungal population, allowing older yeast cells to accumulate over time. Explicitly, older *C*. *neoformans* were shown to persist and accumulate in the spinal fluid of rats and human patients with chronic cryptococcosis^11^. Similarly, old *C*. *glabrata* cells accumulate during chronic infection and when hosts were made neutropenic, younger cells dominated. These findings reinforce the concept that old cells accumulate because they are more resistant to killing by the host neutrophils^10,11^.

Comparisons of transcriptome data from young (0–3 Gen) and old *C*. *glabrata* cells (14 Gen) has yielded significant differences in genes associated with the ergosterol pathway, which were significantly up-regulated in older cells compared to younger cells^9^. This is relevant because FLC and other azoles target Erg11p, a protein encoded by *ERG11* and a rate-limiting step in ergosterol synthesis. Alteration in the ergosterol pathway results in altered susceptibilities to several azoles including FLC as observed in *Saccharomyces cerevisiae*^14^. Besides the ergosterol pathway, genes encoding membrane transporters that are associated with FLC resistance were also upregulated in 14-Gen old *C*. *glabrata* cells^13^. Energy-dependent ABC transporters (ABC-T), one of the two major types of membrane transporters present in yeast, have been implicated in promoting azole resistance in clinical isolates of *C*. *albicans*, *C*. *glabrata*, *C*.*parapsilosis*, and *C*. *dubliniensis*^15^. We have also previously demonstrated that enhanced efflux mediates increased FLC tolerance in old *C*. *glabrata* cells. Specifically, ABC-Ts Cdr1p- and Pdr16p-encoding genes were overexpressed in the old (14-Gen) *C*. *glabrata* cells of two strains^9,13^.

Given that aging pathways may potentially reveal insight in the mechanisms and potential targets for novel antifungal approaches^16^, we sought to characterize replicative aging in *C*. *auris* and specifically investigate if aging was associated with changes in FLC resistance and in changes of other virulence traits.

## Results

### Characterization of *C*. *auris* clinical isolates

We received 11 clinical *C*. *auris* isolates from Centers for Disease Control and Prevention (CDC). These isolates were subjected to antifungal susceptibility testing by measuring their minimum inhibitory concentration (MIC) towards antifungals FLC, 5-flucytosine (5FC), AMB, and micafungin (MF). Seven (S3, S4, S5, S6, S8, S9, and S10) out of 11 isolates had high MICs to FLC (MIC ≥ 64 µg/ml, Table 1), while all the 11 isolates had high 5-FC MICs (MIC ≥ 1 µg/ml, Table 1). In contrast, all the 11 isolates were susceptible to both MF and AMB as evident from data in Table 1.

**Table 1:** Minimum Inhibitory Concentration (MIC) of *C*. *auris* strains.

Strains	MIC (µg/ml)			
	FLC	MF	5FC	AMB
S1	1	0.2	8	0.63
S2	2	0.2	1	0.31
S3	125	0.79	16	0.31
S4	62.5	0.79	4	0.31
S5	500	0.2	8	0.63
S6	500	0.2	8	0.63
S7	4	0.2	1	0.31
S8	250	0.2	8	1.25
S9	250	0.05	>64	0.63
S10	250	0.05	8	0.63
SB	1	0.2	8	0.31

**Figure 1:**
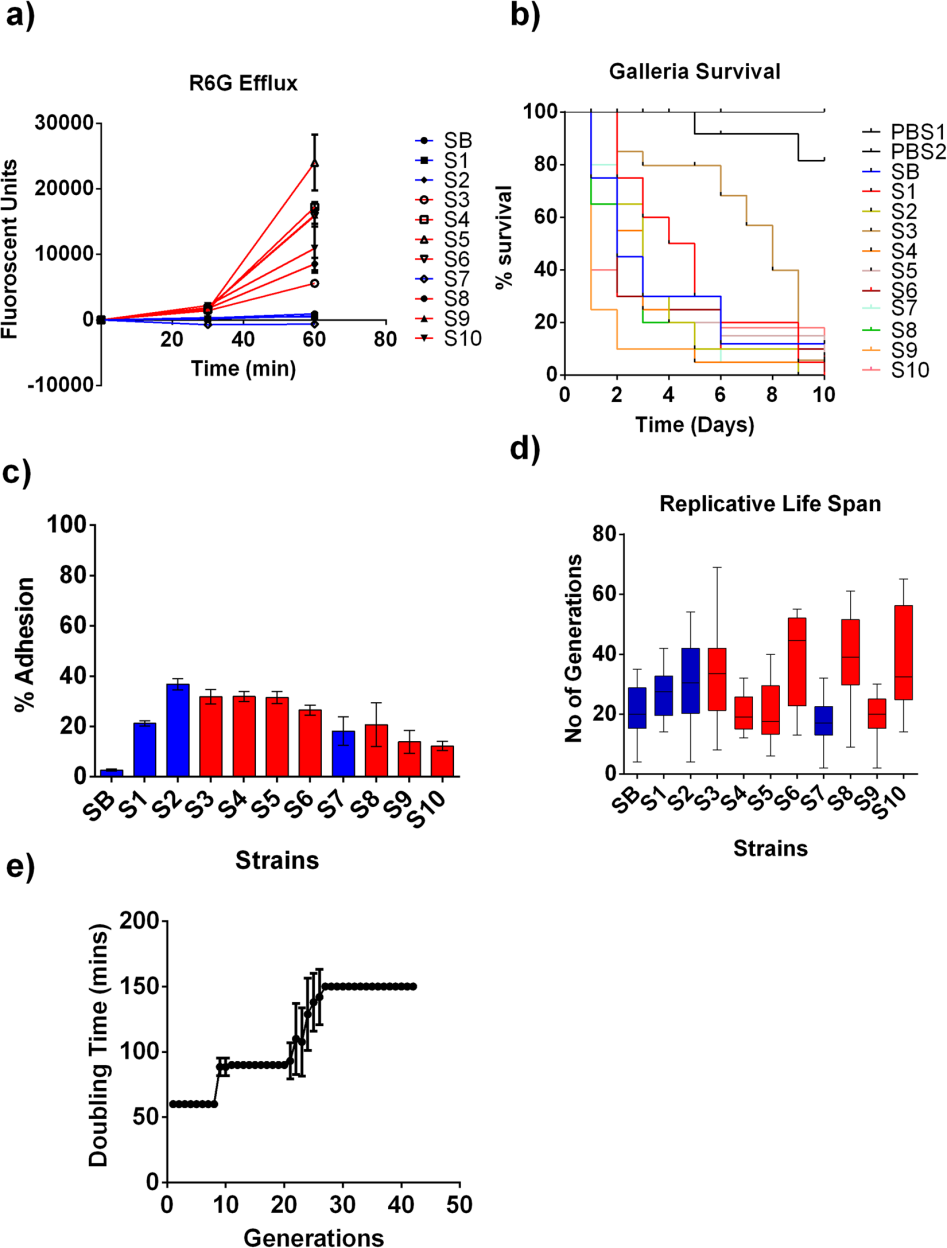
Antifungal Resistance Mechanism and Virulence Phenotypes Observed in the *C*. *auris* Clinical Isolates: (**a**) R6G assay was performed on all the 11 *C*. *auris* strains. Red lines signify FLC resistant isolates, blue lines signify FLC susceptible isolates. R6G assay was done in triplicate, and error bars signify standard deviation. (**b**) *C*. *auris* isolates show variable virulence in *Galleria*. 10^5^ cells from each isolate were injected into 20 larvae (n = 20) and their survival was observed every 24 h for 10 days. Two sets of 20 larvae (labelled in the figure as PBS1 and PBS2) were injected with only 1X PBS as controls. (**c**) Percent adherence of all the 11 *C*. *auris* strains to HeLa cells was measured. Red and blue bars signify FLC resistant and susceptible strains respectively. The assay was done in triplicate and error bars signify standard deviation. (**d**) Representative data of RLS of *C*. *auris* clinical isolates. RLS for each strain was determined for n = 20 cells. The minimum and maximum values of all data are indicated by the ends of the whiskers of box plots. The middle box is the interquartile range divided at the median. The Red and blue bars signify FLC resistant and susceptible strains respectively. (**e**) Change in doubling time of strain S1 was observed with increase in generational age (n = 20). Error bars represent Standard deviation and One-Way Anova was done to determine the significance. Doubling times significantly changed between Gen 1 and Gen 10 cells, Gen 1 and Gen 20, and Gen 1 and Gen 30 (*p < 0.05).

**Figure 2:**
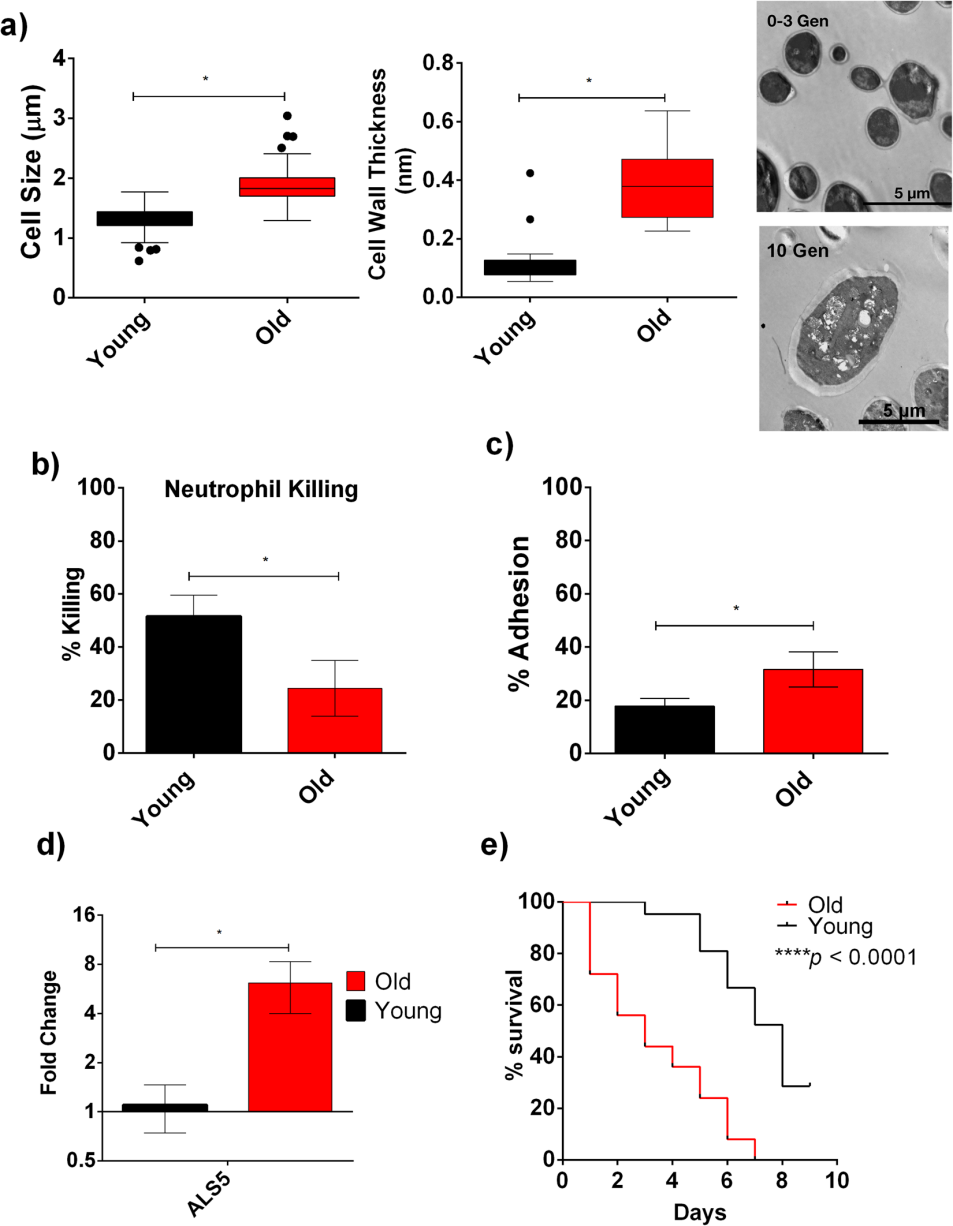
Increased Virulence of 10 Gen Old *C*. *auris* Cells from FLC- Susceptible S1: (**a**) Cell size and Cell wall thickness of the old (10 Gen) cells were significantly greater compared to young (0–3 Gen) cells. Cell wall thickness (n = 23) and Cell Size (n = 150) were measured by ImageJ software from the microscopic images of individual young and old cells. Student’s t-test was performed to analyze the significance (*p < 0.001). Transmission Electron Microscopic (TEM) image of the old (10 Gen) cells of *C*. *auris* showing increased cell wall thickness compared to the young (0–3 Gen) cells. (**b**) Percent Neutrophil-mediated Killing of young (0–3 Gen: black bar) and old (10 Gen: red bar) cells of *C*. *auris*. The assay was done in triplicate and error bars signify standard deviation. Student’s t-test with Welch’s correction was performed to determine the significance; *p = 0.0266. (**c**) Percent adhesion of young (0–3 Gen: black bar) and old (10 Gen: red bar) cells of *C*. *auris*. The assay was done in triplicate and error bars signify standard deviation. Student’s t-test with Welch’s correction was performed to determine the significance; *p = 0.0291. (**d**) qPCR analysis to study the expression of *ALS5* in young and old cells. *ACT1* gene was used as an internal control and the data was normalized to gene expression in young cells. The assay was done in triplicate and error bars signify standard deviation. Multiple t-test was performed using Holm-Sidak method to analyze the significance; *p = 0.016. (**e**) Virulence of young (0–3 Gen) and old (10 Gen) cells of *C*. *auris* in *Galleria*. 20 larvae were infected separately with 10^5^ young and old cells. Log rank test was used to determine the statistical significance.

Both the FLC-resistant and FLC-susceptible *C*. *auris* isolates were subjected to a Rhodamine 6-G (R6G) efflux assay. R6G is a fluorescent dye, the influx and efflux of which is extensively used to study the activities of fungal ABC transporters, which significantly contribute to FLC resistance^17–19^. Increased R6G efflux was observed in FLC-resistant isolates (Fig. 1a, red lines) when compared to the R6G efflux in FLC-susceptible isolates (Fig. 1a, blue lines), whereas import was comparable despite the thickened cell wall (Fig. 2a). Next, virulence of all isolates, including seven FLC-resistant (S3, S4, S5, S6, S8, S9 and S10) and four FLC-susceptible (SB, S1, S2, and S7), was assessed in a *Galleria melonella* infection model. Most *C*. *auris* isolates killed more than 50% of the larvae within 4 days at an inoculum dose of 10^5^ yeast cells (Fig. 1b). Two isolates (FLC-susceptible isolate S1 and FLC-resistant isolate S3), exhibited decreased virulence in *Galleria* (Fig. 1b), when compared to the virulence of FLC-resistant isolate S9 (*p < 0.05 by log-rank test). Virulence of yeast is commonly associated with its ability to adhere to the host cells^20^. Consequently, we assessed adherence of all 11 *C*. *auris* isolates to HeLa cells. Variable adherence was observed among the *C*. *auris* isolates, with isolate SB adhering the least (~3%) and isolate S2 adhering the most (~40%) to HeLa cells (Fig. 1c).

Prior data from our lab showed that *Galleria* virulence and adherence are altered by replicative aging in *C*. *glabrata* and *Cryptococcus neoformans*^9,11^. To investigate age-related phenotypes in *C*. *auris*, the RLS of the 11 clinical isolates had to be first determined. All the isolates showed variable RLS, with median RLS ranging from 18 generations (S5) to 50 generations (S6) (Fig. 1d). Impressive variability of life span, also referred to as stochasticity of RLS, was also noted within same isolates, for example, in strain S10, RLS ranged from 14 to 65 generations. With increase in generational age, the doubling time of the cells increased significantly (Fig. 1e). For example, the doubling time of 1–8 generatiosn old cells from isolate S1 was initially 60 mins but increased to 90 mins in cells aged between 9–30 generations and 150 min in cells above 30 generations old (Fig. 1e).

### Phenotypic variations associated with replicative aging in *C*. *auris*

**Figure 3:**
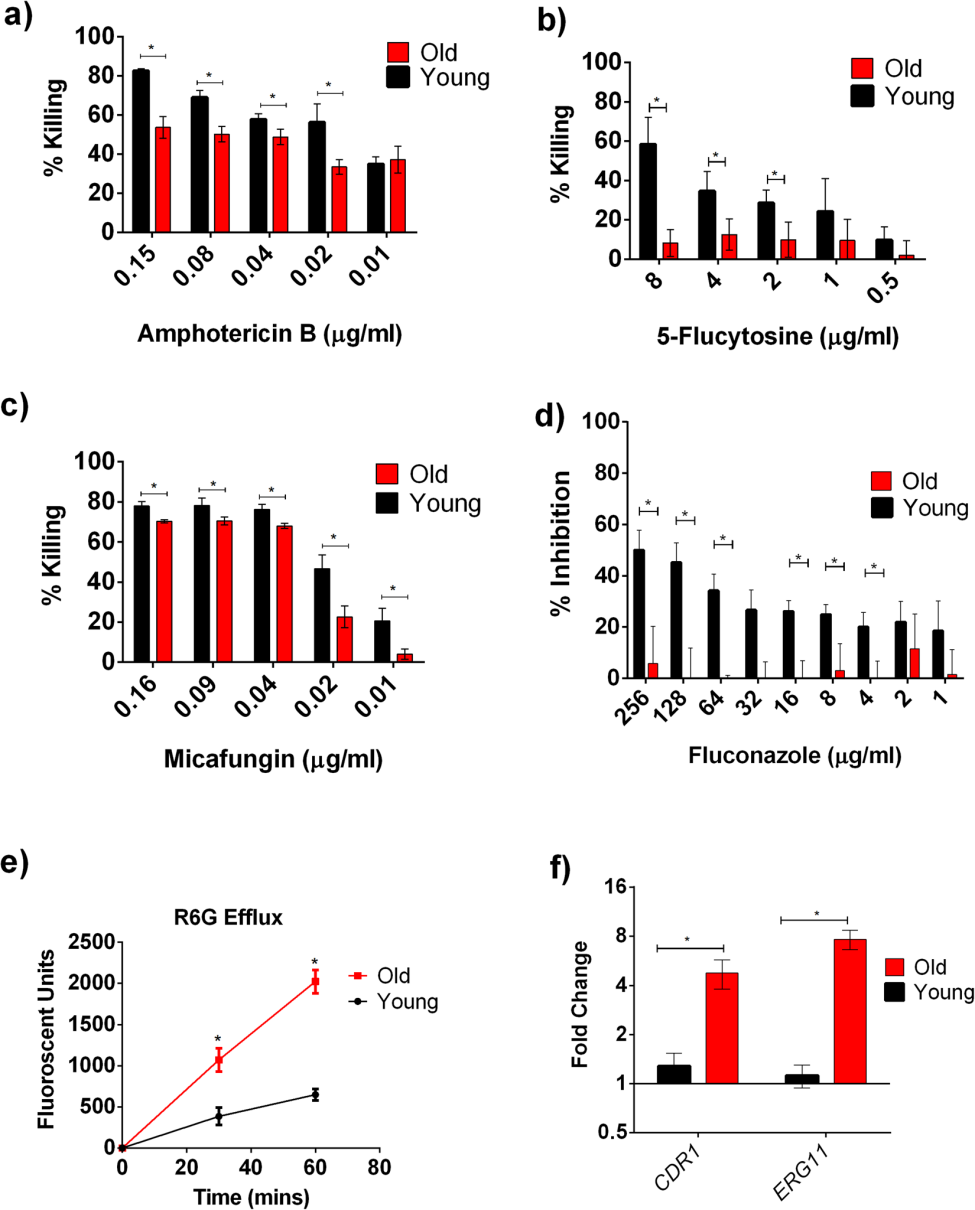
Increased Antifungal Tolerance of 10 Gen Old *C*. *auris* cells from FLC- Susceptible S1 strain: Old (10 Gen) cells are significantly more tolerant to antifungals (**a**) AMB, (**b)** 5FC, (**c**) MF, (**d**) FLC. Black bars signify young cells, while red bars signify old cells. The assays were done in triplicate and error bars signify standard deviation. Multiple t-Test was performed using Holm-Sidak method to analyze the significance; *p < 0.05; (**e**) R6G efflux assay performed on young (black line) and old (red line) in triplicate and error bars signify standard deviation. Student’s t-test with Welch’s correction was performed to determine the significance; *p < 0.05. (**f**) qPCR analysis to study the expression of *CDR1* and *ERG11* in young and old cells. *ACT1* gene was used as an internal control. The data was normalized to the gene expression in young cells. The assay was done in triplicate and error bars signify standard deviation. Multiple t-test was performed using Holm-Sidak method to analyze the significance; *p < 0.05.

*C*. *auris* isolate S1 was chosen to study age-related phenotypic variations between mother and daughter cells. The isolate S1 has a median RLS of 27.5 generations (range 14–42 generations). Important replicative age related phenotypic variations are thickened cell wall and increased cell size that were observed in older generation cells of *C*. *neoformans*^10^, *C*. *glabrata* cells^9^ and in *S*. *cerevisiae*^21^. To test whether these occur in *C*. *auris*, *C*. *auris* clinical isolate S1 cells were aged to 10 generations (defined here as “old cells”) and transmission electron microscopy was performed. Images of old cells demonstrated significant increase in cell wall thickness as well as significant increase in cell size (Fig. 2a). To test whether this increased thickness and size shields old cells from neutrophil-mediated killing, we performed a neutrophil-mediated killing assay using young *C*. *auris* and old *C*. *auris* cells. Young cells were killed significantly more than 10 generation old cells (50.17% vs 24.45% respectively, Fig. 2b). Further, old cells (10 generation) adhered more to the HeLa cells when compared to young cells (31.6% vs 17.83%, Fig. 2c). Increased adherence may result from increased expression of *C*. *albicans* homolog agglutinin-like sequence protein-encoding gene *ALS5*, which was 6-fold up- regulated in old *C*. *auris* cells (Fig. 2d). Als5p is an adhesin protein present in *C*. *auris* that was previously reported to aid in biofilm adhesion in *C*. *auris*^22^. Additionally, increased adherence is often associated with increased virulence, and we observed this in comparing survival of old cells versus young cells in the context of *Galleria* infection (Fig. 2e). Most importantly, old *C*. *auris* cells from FLC-susceptible isolate S1 also acquired significant tolerance to antifungals AMB, MF, 5-FC and FLC (Fig. 3a–d).

### Possible molecular mechanisms of increased FLC tolerance in old *C*. *auris* cells

**Figure 4:**
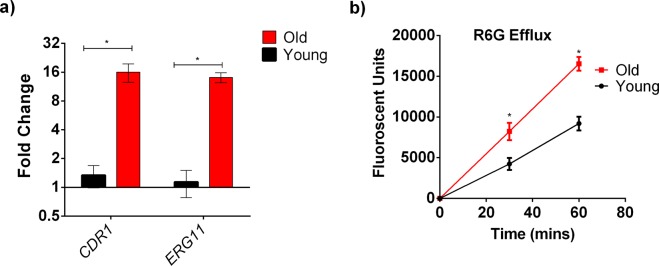
Increased Antifungal Tolerance of 10 Generation Old *C*. *auris* cells from FLC- Resistant S9 strain: (**a**) qPCR analysis to study the expression of *CDR1* and *ERG11* in young and old cells. *ACT1* gene was used as an internal control. The data was normalized to the gene expression in young cells. The assay was done in biological triplicate and error bars signify standard deviation. Multiple t-test was performed using Holm-Sidak method to analyze the significance; *p < 0.05. (**b**) R6G efflux assay performed on young (black line) and old (red line) in triplicate and error bars signify standard deviation. Student’s t-test with Welch’s correction was performed to determine the significance; *p < 0.05.

It is conceivable that increased cell wall thickness in old *C*. *auris* cells could affect FLC import. However, old *C*. *glabrata* were shown to have increased efflux pump activities and FLC resistance, suggesting age-associated increase in efflux may also play a role in *C*. *auris* FLC resistance^13^. We employed the R6G assay to assess efflux pump activities in young and old cells. These data demonstrated comparable influx of RG6 (data not shown), but significant increase in efflux of R6G in the old cells when compared to the young cells (Fig. 3e). R6G is a substrate of ABC transporter Cdr1p, which is linked with azole resistance in *Candida* sp. The expression of both *CDR1* along with the FLC target encoding *ERG11* was observed to be upregulated by 4-fold and 8-fold respectively in old *C*. *auris* cells isolated from FLC-susceptible strain S1 (Fig. 3f). Increased expression of both *CDR1* (16-fold) and *ERG11* (16-fold) was also observed in the old cells isolated from FLC-resistant *C*. *auris* isolate S9 (Fig. 4a). Consistent with these upregulations old cells of S9 also exhibited even more pronounced increased R6G efflux compared to young population (Fig. 4b).

### Gene duplication in old cells may cause increase expression of drug resistant genes

**Figure 5:**
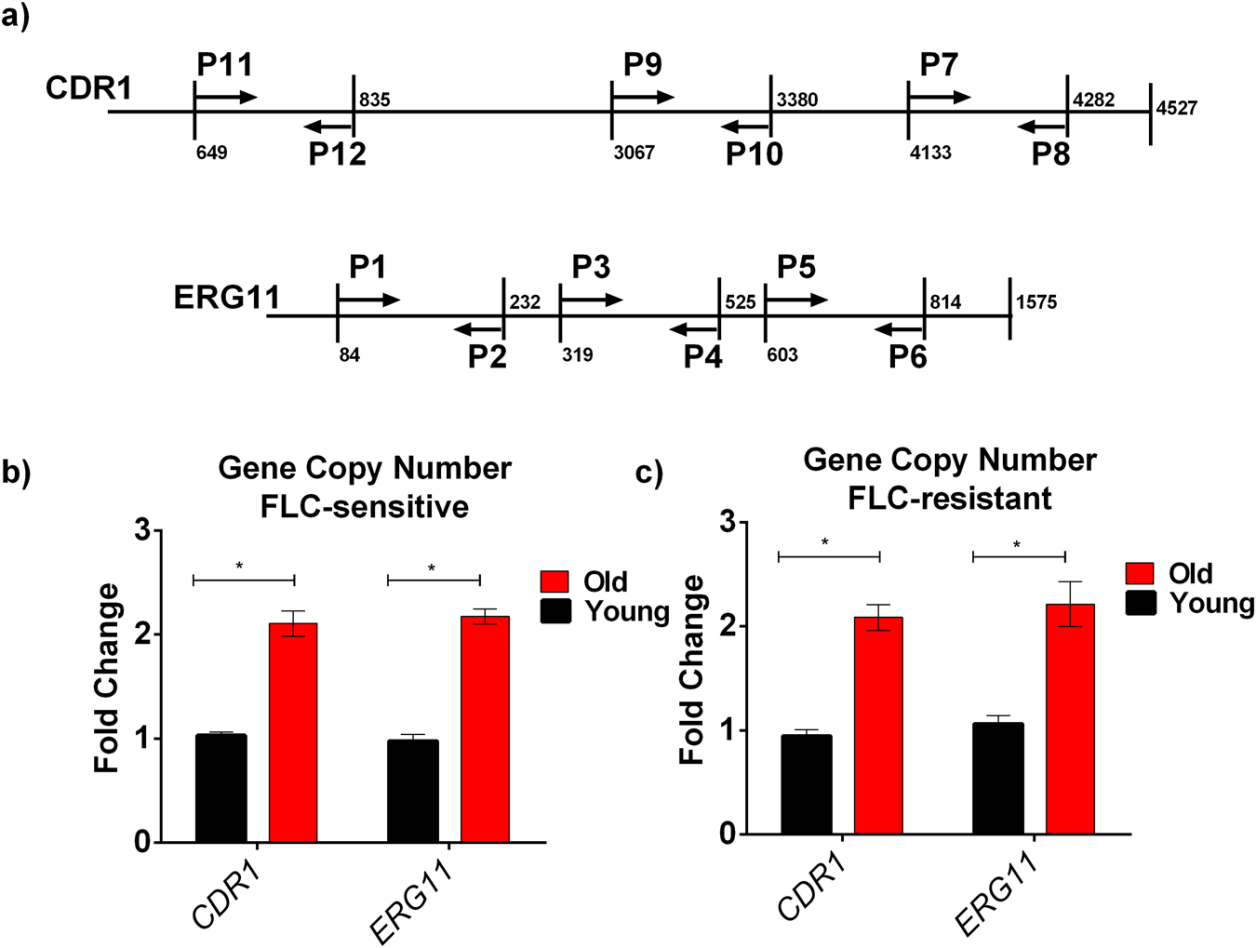
qPCR Analysis to Study the Increase in Copy Numbers of *CDR1* and *ERG11* in Old *C*. *auris* cells isolated from strain S1.: (**a)** Cartoon showing the location of the oligonucleotides used in this analysis. The primers span 3 different regions of each genes. “P” denotes primer. All primers listed in Table S1. Genomic DNA was isolated from both old (10 generation) cells and young (0–3 generation) cells of FLC-sensitive strain S1 **(b)** and FLC-resistant strain S9 **(c)**. Data shows the gene copy numbers of *CDR1* and *ERG11* using oligo sets P7-P8 (*CDR1*) and P5-P6 (*ERG11*) ACT1 was used as a control for the experiment and the data was normalized to the gene copy number of young cells. The assay was performed in triplicate and error bars signify standard deviation. Multiple t-test was performed using Holm-Sidak method to analyze the significance; *p < 0.05.

Although gain-of-function mutations in their respective transcription factors could possibly increase expression of *CDR1* and *ERG11*^23^, this mechanism could not explain upregulation of these genes in old cells, since age-related phenotypes are not inherited by the young cells. Another, mechanism by which *CDR1* and *ERG11* expression could be induced in old *C*. *auris* cells is transient duplication of gene copies in the old cell’s genome. To test this hypothesis, genomic DNAs from both young and old cells from a FLC-sensitive and FLC resistant strains were isolated and subjected to qPCR. Three sets of oligonucleotides (Table S1) were used to span the entire *CDR1* and *ERG11* gene. The oligos were designed in such a manner that they span the entire genes as shown in Fig. 5a. qPCR analysis with these oligos demonstrated that the copy numbers of the *CDR1* and *ERG11* gene doubled in both FLC-sensitive and FLC-resistant strains as evident in Fig. 5b,c that show the results from one oligonucleotide set. Amplification with two other oligonucleotide sets yielded comparable results in both the strains (data not shown). To further investigate the cause of the observed gene duplication, we compared ploidy and DNA content of old and young cells by flow cytometry. No change in total DNA content of young and old cells were observed (Fig. S2).

Besides increased expression of *ERG11* and *CDR1*, increased expression of adhesin encoding gene *ALS5* was also observed in old cells when compared to young cells (Fig. 2d). *ALS5* is an adhesion protein whose function is not associated with *ERG11* and *CDR1*. Of note is that although there was an increased expression of *ALS5*, duplication of this gene in the genome was not observed in older *C*. *auris* cells (Fig. S1).

Most importantly, gene duplication was limited to the older mother cells and disappeared when the old cells were allowed to replicate into a younger cell population (Fig. S3). The old cells were grown into fresh media for 3 h (two doublings). After growth, young and old cells were isolated, and qPCR was performed by obtaining genomic DNA from the young cells obtained by re-growing the 10 generation old cell.

**Figure 6:**
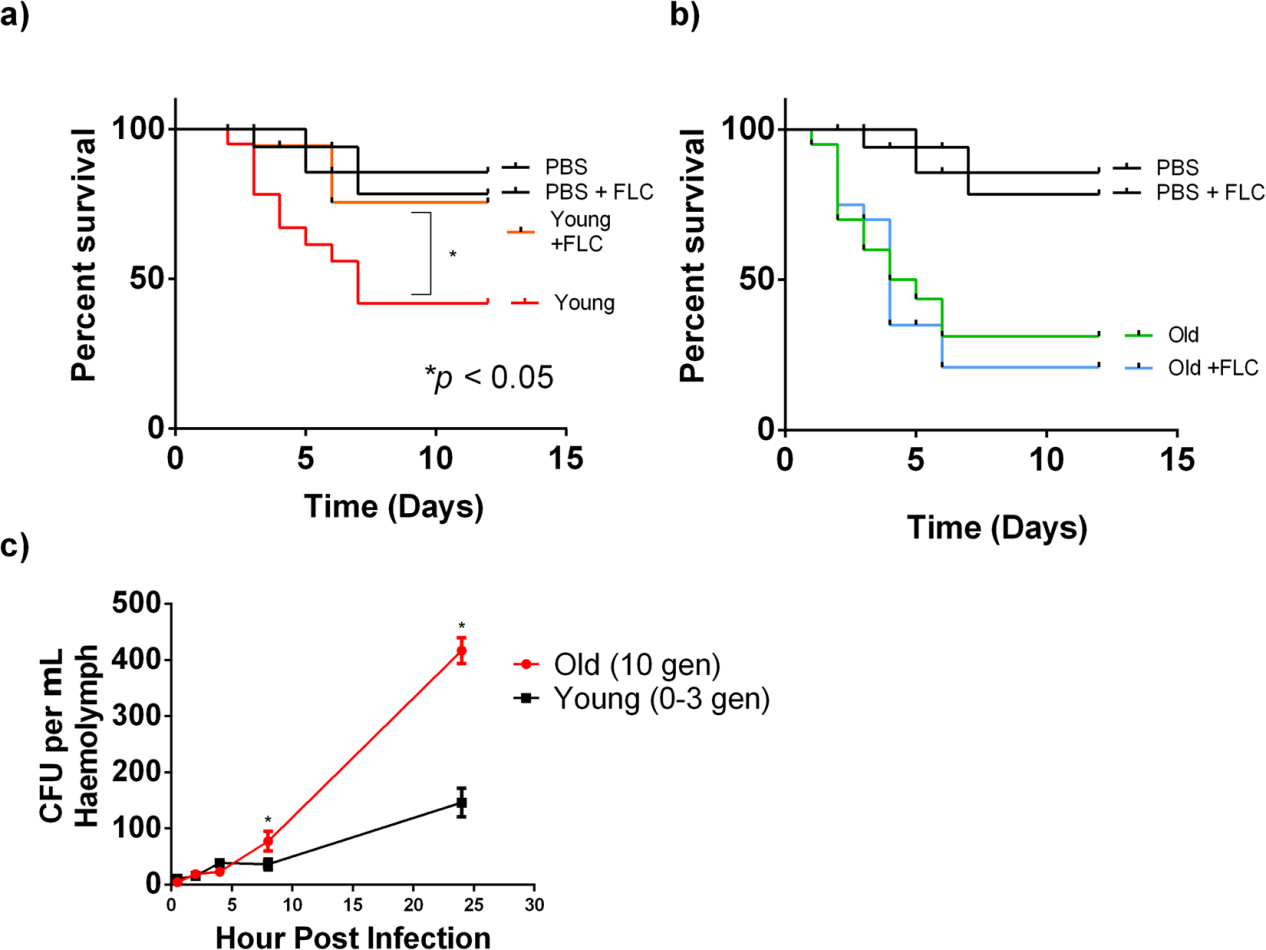
FLC Treatment of *Galleria* infected with Young (0–3 Gen) and Old (10 Gen) cells of *C*. *auris:* 20 larvae were infected separately with 10^5^ (**a**) young and (**b**) old cells. 1 µg/ml of FLC was injected to the larvae infected with young and old population on the same day of infection. Log rank test was used to determine the statistical significance. (**c**) Cells from old (10 Gen) S1 strain were significantly retained more in the haemolymph (*p < 0.05) when compared to young cells at different time points. Haemolymph was collected from 15 larvae over the period of infections (0, 0.5, 2, 4, 8 and 24 h) as described and plated in YPD plates. CFUs were counted and plotted after 48 h of incubation at 37 °C. For each time point, 5 × 10^4^ young and old cells from isolate S1 was infected into 15 larvae. Error bars represent standard deviation in the CFU counts. (n = 15) for each infection time point. Multiple t-test was performed using Holm-Sidak method to determine the statistical significance (*p < 0.05).

The relevance of this finding was further explored in the host environment. Specifically, *Galleria* was infected with old and young *C*. *auris* cells and treated with FLC or PBS. *Galleria* infected with young *C*. *auris* population when treated with FLC survived longer than the *Galleria* infected with young *C*. *auris* and treated with PBS only (Fig. 6a). In contrast, no difference in survival was observed between FLC treated or untreated *Galleria* when *Galleria* was infected with old *C*. *auris*. Here treated or untreated *C*. *auris* exhibited comparable survival (Fig. 6b). Additionally, we assessed whether old cells were resilient in *Galleria*. *Galleria* larvae were infected with either young or old cells from isolate S1, and haemolymph was extracted from the infected larvae at different time points to assess initial clearance. Initially, larvae infected with young cells were able to clear a portion of the young cells from haemolymph whereas there was no clearance seen in the haemolymph of larvae infected with old cells. In fact, old cells were able to replicate faster than they were cleared from the haemolymph (Fig. 6c).

## Discussion

This study is the first to investigate replicative aging in multi-drug resistant *C*. *auris* clinical isolates and yields important results that inform our understanding about the emergence of azole resistance in this important pathogenic yeast. *C*. *auris* constitutes a threat to the medical community as the majority of clinical isolates obtained from patients are resistant to multiple anti-fungals^4^. Indeed 70% of the isolates used in this study exhibited elevated MICs to FLC. Additionally, FLC resistance emerges rapidly in patients being treated with azoles^24^. Why this species rapidly emerged as multidrug resistant is not understood and may not only be the result of exposure to azole prophylaxis. Resistant *C*. *auris* isolates have increased R6G efflux when compared to the susceptible isolates in our collection. R6G is frequently used to study efflux activities of ABC-transporters, and increased efflux significantly corelates with azole resistance in various *Candida* sp.^18,19,25^. Similarly, our data is supported by other data that also suggests that increased efflux activities cause the observed FLC resistance in *C*. *auris*^2^.

Besides FLC, *C*. *auris* isolates exhibit high MICs to 5-FC, an antifungal which is at times used synergistically with FLC to treat candidiasis^26^. In this study we found that *C*. *auris* undergoes replicative aging similar to *Cryptococcus neoformans* and *C*. *glabrata* with increasing doubling times over time^9^. As expected, *C*. *auris* strains of different clonal backgrounds also exhibit variable RLS, consistent with the observed RLS variability of other yeast strains^9,11^.

During the process of replicative aging in *Cryptococcus neoformans* and *C*. *glabrata*, phenotypic traits emerge that promote selection of older cells and cause them to accumulate and persist in the hosts during chronic infections. These older cells are more resilient to antifungal and neutrophil killing^9^. Hence, we were interested in investigating if replicative-aging-related resilient phenotypes also emerge in *C*. *auris*. Older *C*. *auris* cells exhibit thickened cell wall and increased adhesion to HeLa cells. *C*. *auris* adhesin protein Als5p shares 34% homology with N-terminal end of *C*. *albicans* Als5p, which is the extracellular binding portion of the protein^27^. Our data demonstrates 8-fold increased expression of *C*. *auris ALS5* in the older population. Als5p in *C*. *auris* has recently been described as an important adhesin protein that aids in biofilm adhesion^22^. Als5p belongs to agglutin-like sequence family of protein and exhibits high homology to Als3p, the latter is an important adhesin protein, which has been incorporated in anti-candida vaccine^28^, where antibodies against Als3p predict vaccine efficacy *in vivo*^29^. Besides this, increased adherence in older *C*. *auris cells* to host cells supports the concept that aging involves remodeling of the cell wall, which contributes to the increased virulence of older *C*. *auris* cells in *vivo* in the *Galleria* infection model.

We found 80% of 10 generation old cells from FLC- sensitive isolates survived at FLC concentrations of 256 µg/ml and 64 µg/ml respectively, although the 0–3 generation young daughter cells of the FLC-sensitive isolates exhibited FLC MICs around 1 µg/ml. This is consistent with our observation that FLC treatment failed to cure in the *Galleria* infection model experiment when older *C*. *auris* cells were administered. Increased tolerance to FLC in the older *C*. *auris* cells may result from two mechanisms. First, old cells are retained significantly more in the haemolymph of *Galleria*, and second, the combination of overexpression of drug resistant genes *CDR1* and *ERG11*, both of which *w*ere observed. Older *C*. *auris* cells exhibited increased efflux of R6G, which is a substrate for Cdr1p ABC transporter. This is consistent with the increased ABC transporter mediated efflux observed in old *C*. *glabrata* cells^13^. Therefore, drugs such as Sir2p agonist that modify the RLS of a *C*. *auris* population should be investigated because studies suggest that they constitute a potential class of novel antifungal drug targets, which enhance antifungal efficacy by shifting lifespan and vulnerability of the fungal population^16^.

Cdr1p and Erg11p mediated drug resistance is extensively studied in *C*. *albicans*^18,19,30^. Both Cdr1p and Erg11p can be induced by acquiring gain-of- function mutations in the transcription factors Tac1p and Upc2p respectively in response to antifungal stress^31,32^. Our results strongly suggest that a transient gene duplication occurs and the increased copy number of *CDR1* and *ERG11* results in the increased expression of *CDR1* and *ERG11* in the older *C*. *auris* cells. Gene copy numbers were analyzed by qPCR, which can reliably quantify gene copy numbers^33^. Three sets of oligonucleotides were used in two independent strains in this study to span the entire *CDR1* and *ERG11* genes. These data show that whole *CDR1* and *ERG11* genes are duplicated in older cells and more importantly only transiently, as the gene duplication is lost when the older cells are allowed to replicate to produce young daughter cells. It is conceivable that the stress of continuous replicative aging in cells of advanced generational age may select for the observed duplication event. Consistent with that interpretation is the data in S. *cerevisiae*, which demonstrates that the RLS in young cells obtained from old cells is reset and that young cells do not inherit materials from old mother cells^34^. Whether this gene duplication is a result of a chromosomal duplication event cannot be concluded from our data. At this point the available whole genome sequencing data in *C*. *auris* is not granular enough to determine chromosome numbers and assign genes to individual chromosome locations. However, it is known that aneuploidy can, despite being a severe burden on the cell, confer a selective advantage. Specifically, in *C*. *glabrata* as well as in *C*. *neoformans* aneuploidy has been associated with hetero-resistance to FLC^35^. In *C*. *albicans*, *CDR1* and *ERG11* are located in two different chromosomes (chromosomes 3 and 5 respectively). Without the precise knowledge of chromosomal locations of *CDR1* and *ERG11* we cannot know if these two genes are on the same chromosome in *C*.*auris*. It is noteworthy that *ALS5* gene copy does not change with aging. Causal relationship between genomic instability and replicative aging has been suggested^36–38^, but still remains incompletely understood. Most data to date has investigated genomic instability at the ribosomal DNA (rDNA) locus in the process of aging^39^. Chromatin structure constitutes a key modulator of aging in yeast^40^ and previous data indicates global loss of histones from all regions of the yeast genome during replicative aging. Loss of histones results in an open chromatin conformation that is associated with globally increased gene expression and accumulation of DNA breaks^41^. Defects in homologous recombination can lead to elevated levels of DNA breaks, translocations, loss of heterozygosity (LOH), and amplifications, in older yeasts^42^. Further studies need to be done in *C*. *auris* to establish chromosome numbers, and extent of genomic instability and gene duplications that occurs in replicative aging, and it will be important to determine at what generational age it occurs, and how many generations it takes to correct the duplications. Azole resistance via chromosomal duplication are extensively studied in *C*. *albicans*, *C*. *glabrata* and *Cryptococcus neoformans*^43–45^, but they have not been linked to replicative aging. For example, the left arm of chromosome 5 that contains *TAC1* and *ERG11* duplicates causing increased azole resistance in *C*. *albicans*^43^.

Besides increased FLC resilience, old *C*. *auris* cells showed increased tolerance to MF similar to the observed phenotype in old *C*. *glabrata* population^13^. MF targets cell wall, and the thickened cell wall in the older population of *C*. *auris* cells may increase the target drug ratio and explain the only slightly increased MF resistance, which may not be clinically relevant. Additionally, increased tolerance to AMB and 5FC was also observed in old *C*. *auris* cells consistent with the findings in the old *C*. *glabrata* population^13^. In *C*. *glabrata*, old cells exhibit decreased ergosterol content, which may explain increased AMB resilience in old cells^9,13^ and hence future studies will have to determine, the ergosterol content in old *C*. *auris* cells.

This study demonstrates for the first time that replicative aging results in transient duplication of genes that are relevant for FLC resistance. It will be important to acquire complete chromosome maps and further investigate this age-related genomic instability and assess if it remains permanent in some daughter cells. As other traits also promote selection of older cells this epigenetic phenotype of drug resistance could be highly relevant and may have contributed to the rapid emergence of FLC resistance in this species even without extensive exposure to azoles.

## Materials and Methods

### Strains and culture conditions

11 *C*. *auris* strains were obtained from CDC with proper Material Transfer Agreement (MTA). The strains were cultured in Yeast Peptone Dextrose (YPD) media (Difco) and incubated at 37 °C. 30% glycerol stocks were made for each strain and stored in −80 °C.

### Antifungal susceptibility testing

Minimum Inhibition Concentration (MIC) assay was performed on all the 11 *C*. *auris* strains following previously published protocol^46^. Briefly, the *C*. *auris* strains were cultured in YPD at 37 °C for overnight, and 0.1 OD of the cultured cells were used as inoculum for MIC the next day. Antifungals fluconazole (FLC), 5- flucytosine (5-FC), amphotericin B (AMB), and micafungin (MF) were two-fold serially diluted in RPMI 1640 media (GE Life Sciences) with 25 mM HEPES (pH 7.0) in a flat bottom 96-well plate (Costar). The plates were incubated for 48 h at 37 °C without shaking. In the plate, one column without any antifungal and one column without cells were used as controls for the assay. MICs were performed in biological triplicate and the average value of the triplicate is reported.

### Replicative life span (RLS)

RLS was performed as described previously in *C*. *glabrata* and *Saccharomyces cerevisiae*^9,47^. Briefly, 20 virgin cells were arrayed in a straight line in YPD plates, and new buds from the virgin cells were separated using a 25 µm needle (CoraStyles) under a tetrad dissection microscope (Zeiss) at 250X magnification. The plates were incubated at 37 °C at the end of each budding event, and budding was monitored every 1 h. The cells that did not bud for 24 h were considered dead.

### Isolation of old and young cells

Isolation of old and young cells were performed as previously described^9,11^. Briefly, *C*. *auris* cells from isolates S1 and S9 were grown in Synthetic Dextrose (SD) media (6.7 g Bacto Yeast Nitrogen Base without aminoacids and 20 g glucose/litre) overnight. Next day, 0.1 OD of overnight cells were exponentially grown for 8 h, and 10^8^ cells were washed three times with 1X PBS and labelled with 4 mg/ml of Biotin (Sulfo-NHS-LC-Biotin, Pierce, Rockford, IL) for 30 mins at room temperature. Next, the cells were washed three times in 1X PBS, and were grown for 7 generations (~7–8 h) in 50 ml SD media in 250 ml flask. After growth, the cells were washed three times with 1X PBS, and counted in hemocytometer. All the cells were resuspended at a concentration of 10^7^ cells per 90 µl of 1X PBS and incubated with 10 µl of streptavidin conjugated magnetic microbeads (Miltenyi Biotec, Auburn, CA) per 10^7^ cells for 30 mins at 4 °C. After incubation, the cells were washed three times in 1X PBS and resuspended at a concentration of 10^8^ cells per 500 µl of 1X PBS. The cells were subjected to LS magnetic column (Miltenyi Biotec), which retained the cells conjugated with biotin-streptavidin in presence of a magnetic field and allowed the non-labelled cells to pass through. The biotin-streptavidin labelled cells were recovered by removing the magnetic field. This fraction containing the 7 generation old cells, was then washed three times in 1X PBS and grown in SD media for three more generations (~3–4 h). After growth, the cells were subjected to LS column as described above to isolate 10 generation old cells. In this publication old cells refers to 10 generation old cells. At 10 generations S1 cells have completed about 36% of their median RLS (27.5 generations). The purity of the old cell population was confirmed by staining the streptavidin labelled cells with Fluorescein Isothiocyanate (FITC), and the population was at least 90% pure.

### Transmission electron microscopy

Young and old cells were isolated from *C*. *auris* isolate S1 and resuspended in 5% glutaraldehyde and fixed for 2 h at room temperature. Imaging was done by Stony Brook University microscope facility following previously published protocol^9^. Cell wall thickness (n = 23) and Cell Size (n = 150) were measured by ImageJ software from the microscopic images of individual young and old cells.

### Antifungal killing assay

Antifungals FLC, 5-FC, AMB, and MF were serially diluted in 100 µl RPMI 1640 media at concentrations of 256 to 1 µg/ml, 8 to 0.5 µg/ml, 0.15 to 0.01 µg/ml, and 0.15 to 0.01 µg/ml respectively. Both young and old *C*. *auris* cells from isolate S1 were isolated as described above and resuspended in RPMI media. 100 µl of 10^4^ cells/ ml were plated in flat bottom 96-well plate containing 100 µl of serially diluted antifungals, to achieve a final cell concentration of 5 × 10^3^ cells/ml in each well. The plates were incubated for 2 h at 37 °C without shaking. After incubation 100 µl of drug treated cells were plated in 25 ml YPD agar plates and incubated at 37 °C for 48 h. Cells without antifungal treatment were used as controls. The percent killing was calculated by comparing the number of colony forming units (CFUs) of antifungal treated cells and the number of CFUs in the control (non-treated cells). The experiment was performed in triplicate.

### Neutrophil-mediated killing assay

The neutrophils were isolated as described previously^48^. The neutrophil-mediated killing assay was performed as before^49^. Briefly, 10^5^ neutrophils were diluted in RPMI 1640 media and incubated with 1% human serum for opsonization and were incubated for 1 h at 37 °C with 5% CO_2_ in flat-bottom 96 well plate. Young and old (10 gen) cells from *C*. *auris* isolate S1 were isolated and added to the wells containing neutrophils at a fungus to neutrophil ratio of 1:10. The wells with no neutrophils were used as controls. The plates were incubated for 1 h at 37 °C with 5% CO_2_ to allow phagocytosis and then were washed three times with RPMI 1640 media. One set of neutrophil-fungal cells after phagocytosis was immediately lysed with sterile water and plated in YPD media to use as a phagocytic control. These cells were subjected to no killing by neutrophils. Other set of neutrophil-fungal cells were incubated for 2 h at 37 °C with 5% CO_2_ to initiate neutrophil killing. After 2 h, the neutrophils were lysed and 100 µl of the reaction mixture was plated in YPD media and incubated at 37 °C for 48 h. Percent killing was calculated by comparing the CFUs between 2 h neutrophil killed cells and non-killed cells. The experiment was performed in triplicate.

### Infection model

*Galleria mellonella* infection model was carried out as previously described using Hamilton Syringe^9,50^. *G*. *mellonella* caterpillars in the final instar larval stage were obtained (Vanderhorst Wholesale Inc., St. Mary’s, Ohio) and were stored in the dark. The larvae were used within 7 days from the day of shipment. Larvae employed in all assays were randomly chosen. Briefly, 10^5^ young and old (10 gen) from isolate S1, or exponentially growing *C*. *auris* cells from all isolates were injected into each larva. For each condition, 20 larvae were used, and larvae injected with only 1X PBS was used as control. Additionally, in a separate experiment, infected larvae were treated with 1 µg/ml of FLC, on the same day of infection to study the effects of FLC treatment *in vivo*. In an independent experiment, retention of young and old *C*. *auris* cells in haemolymph of *Galleria* larvae was analyzed as previously described^9^ by infecting the larvae with 5 × 10^4^ young and old cells from isolate S1. Haemolymph was extracted after 0, 0.5, 2, 4, 8 and 24 h of infections, plated on YPD agar plates, and CFUs were counted after 48 h incubation at 37 °C. For each time point, haemolymph from 15 (n = 15) larvae was collected.

### Rhodamine 6G efflux assay

Rhodamine 6G (R6G) efflux assay was performed as described previously^18^. Briefly, 10^8^ young and old (10 gen) cells from isolate S1 or exponentially growing cells of all isolates were starved for 2 h in presence of 5 mM deoxy glucose (Sigma-Aldrich) at 37 °C with shaking and were washed three times with 1X PBS. R6G (Sigma-Aldrich) was then added to the washed cells at a final concentration of 10 µM and incubated for 30 mins at 37 °C with shaking to initiate R6G import. After incubation, the cells were washed three time in 1X PBS, and efflux was initiated by adding 2% glucose. Samples were collected every 30 mins, and the fluorescence of the supernatants were measured at excitation and emission wavelengths of 525 nm and 555 nm respectively. The assay was performed in triplicate.

### Analysis of gene duplication and gene expression with qPCR

RNA was isolated from young and old (10 gen) *C*. *auris* cells from both isolates S1 and S9 using Qiagen RNEASY Kit following manufacturer’s protocol and quantified in a nano drop (BioSpectometer, Eppendorf). A260/280 ratio of 2.0 or above was considered as pure RNA.

For gene expression quantification, 450 ng of RNA was subjected to DNAse (ThermoScientific) treatment following manufacturer’s protocol. qPCR was performed using Power SYBR® Green RNA-to-CT™ 1-Step Kit (Applied Biosystem) following manufacturer’s protocol. Oligonucleotides used for qPCR are listed in Table S1. Housekeeping gene *ACT1* was used as a control for qPCR, and the data was normalized to the gene expression in the young cells. 2^−ΔΔCt^ method was used to calculate the fold change^51^.

For gene copy number quantification, genomic DNA was isolated from both young and old *C*. *auris* cells from both isolates S1 and S9 by using previously published protocol^52^. Only 50 ng of DNA was used to analyze gene copy number with qPCR using Power SYBR Green (Applied Biosystem) following manufacturer’s guidelines. *ACT1* gene was used as a control. 2^−ΔΔCt^ method was used to calculate the change in copy number^51^.

### Cell adhesion assay

Cell adhesion assay was performed as previously described^53^. Briefly, HeLa cells were grown in DMEM (GE Life Sciences) media supplemented with 10% Fetal Bovine Serum (FBS), and 10^4^ cells were added into flat- bottom 96 well plate and were incubated at 37 °C with 5% CO_2_ for 1 h for the HeLa cells to adhere to the wells. After incubation, 10^4^ young and old (10 gen) from isolate S1 or exponentially growing *C*. *auris* cells from all isolates were added on to the HeLa cells and incubated at 37 °C with 5% CO_2_ for 1 h for the yeast cells to adhere to the HeLa cells. The non-adherent *C*. *auris* cells were removed by washing five times with fresh DMEM media. After washing, the adherent *C*. *auris* cells were recovered by lysing the HeLa cells with 0.1% Triton X100 and plating in YPD media. The plates were incubated at 37 °C for 48 h and CFUs were counted. As control, 10^4^ *C*. *auris* cells were directly plated in the YPD media and incubated at 37 °C for 48 h and CFUs were counted. Percent adherence was calculated by comparing the CFUs of the HeLa treated cells with the control. The assay was done in triplicate.

### Flow Cytometry (FACs) Analysis of DNA Content

DNA content of both young and old *C*. *auris* cells (S1) were analyzed as described before^54^. Briefly, the young and the old cells were subjected to permeabilization and fixation via addition of 1 ml chilled 70% ethanol for 2 h at 4 °C. The fixed cells were then washed three times with FACs buffer (0.2 M Tris-HCl (Sigma-Aldrich), pH 7.4, 20 mM EDTA (Sigma-Aldrich) and incubated for 2 h at 37 °C with 1 mg/ml RNase A (Sigma-Aldrich). After RNase treatment, the cells were washed three times with 1X PBS and stained overnight at 4 °C with 100 µl of Propidium Iodide (PI, Sigma Aldrich), at a final concentration of 50 µg/ml of PI. After adding 900 µl of 1X PBS the next day, FACs analysis of the DNA content was done using a FACSCalibur analyzer (Becton-Dickinson). A total of 50,000 cells of both young and old population were analyzed.

### Statistics

All statistics were done in Graph Pad Prism 6.0. The specific statistical test performed are listed in the figure legends.

## Supplementary information

Supplementary Information

## Data Availability

All data required to evaluate and understand the article are included. If additional data is required, the data are available on request from the correspondence author.
