# Supplementary material for: Gene Duplication Associated with Increased Fluconazole Tolerance in Candida auris cells of Advanced Generational Age
Source: Sci Rep. 2019 Mar 25;9:5052. doi: 10.1038/s41598-019-41513-6 (PMC6434143; doi:10.1038/s41598-019-41513-6)
Supplement: Supplementary file 1 — Supplementary Information [file 41598_2019_41513_MOESM1_ESM.docx]

**Supplemental Material**

**Gene Duplication Associated with Increased Fluconazole Tolerance in Candida auris cells of advanced generational age**

**Somanon Bhattacharya1, Thomas Holowka1,+, Erika P. Orner1,2, and Bettina C. Fries1,2,***

**Table S1: List of Oligonucleotides Used**

| **Oligo Name** | **Forward (5’-3’)** | **Reverse (5’-3’)** | **Used in** |
| --- | --- | --- | --- |
| **ACT1** | TCCTCTCAGTCGTCCGCTAT | CTTCATGGAAGATGGGGCTA | **Control (gene copy number + gene expression)** |
| **ERG11S1** | GTGCCCATCGTCTACAACCT  **(P1)** | TCTCTCTGCACAGCTCGAAA  **(P2)** | **Gene copy number + gene expression** |
| **CDR1S1** | GCCAGGTTTCTGGATTTTCA  **(P7)** | GGCCACAAGTTTGACCACTT  **(P8)** | **Gene copy number + gene expression** |
| **ERG111S2** | GCTAAGCTTGCGGATGTTTC  **(P3)** | TTGGGAGCAAGCTTTGAAGT  **(P4)** | **Gene copy number** |
| **CDR1S2** | CCAACCACGGTCAAGCTATT  **(P9)** | ACCTCCAACATCCATTCAGC  **(P10)** | **Gene copy number** |
| **ERG11S3** | CAAGTCGTTGATGGGTGATG  **(P5)** | GAACGATGTCACCGGTCTTT  **(P6)** | **Gene copy number** |
| **CDR1S3** | ACGGTTTTCACATTGGCGAA  **(P11)** | CTTCACCCCTGTTTTGAGGC  **(P12)** | **Gene copy number** |
| **ALS5** | CCTTCTGGATCGGACACAGT | AGTTGTGGTGGAGGAACCAG | **Gene Expression** |

**Supplemental Figure S1. *ALS5* gene duplication.** Genomic DNA was isolated from both old (10 generation) cells and young (0-3 generation) cells of FLC-sensitive isolate S1. Data shows the gene copy number of *ALS5* between young and old generation cells. *ACT1* was used as a control for the experiment and the data was normalized to the gene copy number of young cells. The assay was performed in triplicate and error bars signify standard deviation. Multiple t-test was performed using Holm-Sidak method to analyze the significance; no significant change in copy number between young and old cells was observed

**Supplemental Figure S2. FACs analysis of DNA content.** DNA content analysis of old (10 gen) and young (0-3 gen) of *C. auris* isolate S1. The assay of performed in triplicate. O1, O2, and O3 denote DNA content from triplicate preparation of old (10 gen) cells, while Y1, Y2, and Y3 denote DNA content from triplicate preparation of young (0-3 gen) cells.

**Supplemental Figure S3. Gene duplication of *ERG11* and *CDR1* is not inherited by the young cells from old mother cells.** Old (10 gen) cells from *C. auris* isolate was further grown for two doublings (3 h), and young cells from this old population (denoted here as Young_From_Old) were isolated. Genomic DNA was isolated from these young cells. Data shows the comparison of gene copy numbers of *CDR1* and *ERG11* between young cells (0-3 gen) and Young_From_Old cells. *ACT1* was used as a control for the experiment and the data was normalized to the gene copy number of young cells. The assay was performed in triplicate and error bars signify standard deviation. Multiple t-test was performed using Holm-Sidak method to analyze the significance; no significant change in copy numbers of both genes between young and Young_From_Old cells were observed.
